# Impact of problematic mobile phone use and insufficient physical activity on depression symptoms: a college-based follow-up study

**DOI:** 10.1186/s12889-019-7873-z

**Published:** 2019-12-05

**Authors:** Haibo Xie, Shuman Tao, Yukun Zhang, Fangbiao Tao, Xiaoyan Wu

**Affiliations:** 1 Department of Maternal, Child & Adolescent Health, School of Public Health, Anhui Medical University, 81 Meishan Road, Hefei, 230032 Anhui Province China; 2 Department of Dermatology, The First Affiliated Hospital of Anhui Medical University, Hefei, China; 3 Department of Nephrology, The Second Hospital of Anhui Medical University, Hefei, China; 4 Anhui Provincial Key Laboratory of Population Health & Aristogenics, Hefei, China

**Keywords:** Problematic mobile phone use, Physical activity, Depression, College students

## Abstract

**Background:**

Insufficient physical activity (IPA) and mobile phone dependence are common coexisting behaviors among college students. However, the impact of the synergies between the two behaviors on depression has yet to be validated. Therefore, this study evaluated independent and interactive associations of problematic mobile phone use (PMPU) and IPA with depression symptoms and increased depressive symptoms among Chinese college students.

**Methods:**

In total, 2134 college students participated in this follow-up study, which was conducted between June 2014 (wave 1) and December 2014 (wave 2) at Anhui Medical University. The Self-rating Questionnaire for Adolescent Problematic Mobile Phone Use and the Center for Epidemiologic Studies Depression Scale were used to assess PMPU and depression symptoms, respectively. Physical activity (PA) was assessed with a reliable question from the Youth Risk Behavior Survey. Joint effects of PMPU and PA were calculated, and increased depressive symptoms were assessed. We used multivariable-adjusted odds ratios (OR) with 95% confidence intervals (CI) to evaluate associations between depression symptoms and PMPU, IPA, and the PMPU/IPA joint effect, estimated by binary logistic regression models.

**Results:**

PMPU and high PMPU/IPA joint effect scores were significantly associated with depression symptoms in waves 1 (*OR* 7.36, 95% *CI*: 5.09, 10.66) and 2 (*OR* 3.74, 95% *CI*: 2.56, 5.48). IPA was significantly associated with depression symptoms in wave 1 (*OR* 1.40, 95% *CI*: 1.09, 1.79) but not wave 2 (*OR* 1.24, 95% *CI*: 0.95, 1.62). PMPU and high PMPU/IPA joint effect scores were also significantly associated with increased depressive symptoms (*OR* 2.36, 95% *CI*: 1.55, 3.60).

**Conclusions:**

These results suggest that PMPU is an important factor for depression in college students, and IPA may be a synergistic factor.

## Background

Depression is one of the most prevalent mental health disorders worldwide [1], and has become a global public health burden. Given the context of life transition, transitional-age youth are uniquely vulnerable to mental health problems such as depression [2]. There is some evidence that lifestyle-related risk factors may contribute to depression; for example, screen time exposure [3], unhealthy diet [4], sedentary lifestyle, stressful events [5], physical activity (PA) [6], and sleep problems [7]. However, these associations may be explained by some common mechanisms.

Physical inactivity has also been identified a major public health problem. The World Health Organization estimated that 3.2 million deaths globally are attributable to physical inactivity [7]. Physical inactivity refers to insufficient PA (IPA) in daily life, or less than 150 min of moderate PA per week [8]. However, global data suggest that most youth are not meeting recommended PA levels [9, 10]. It is presumed that PA participation declines over the life course, particularly in adolescence. A systematic review that included 26 studies from around the world found the mean percentage of PA among adolescents had decreased by 7.0% per year during the last decade [11]. Increasing evidence suggests that physical inactivity is associated with increased risk for mental health problems, such as depression [12, 13] and anxiety [14, 15].

With the development and popularization of modern science and technology worldwide, the widespread use of mobile phones is another problem associated with IPA. In 2018, there were more than 7.8 billion mobile phone users worldwide [16]. Problematic mobile phone use (PMPU) is a new term that is defined as an inability to control mobile phone use time, resulting in negative consequences in daily life such as depression [17–19].

IPA and mobile phone dependence are common coexisting behaviors among college students, and previous studies have verified their respective associations with mental health. However, there is a paucity of research on the extent to which the two behaviors combined increase the risk for health problems. The present study hypothesized that IPA and PMPU would be simultaneously associated with depression symptoms, and would have an interactive effect on depression symptoms among college students. The findings are expected to improve understanding of behavioral and emotional problems among college students.

## Methods

### Participants

This two wave follow-up study was conducted between June 2014 and December 2014 at Anhui Medical University, China. All freshmen were invited to participate in this study, and questionnaires [3] were distributed before their class started. Students participated with their student ID. In total, 2835 college freshmen were enrolled in this study in June 2014 (wave 1), and 2616 valid questionnaires were collected. Participants who responded to the wave 1 questionnaire were recruited for the follow-up (wave 2) 6 months later (December 2014); 249 students dropped out. Overall, 2134 students (43% males, 57% females) provided complete data for waves 1 and 2. The present study was conducted according to the guidelines laid down in the Declaration of Helsinki. All procedures involving human subjects were approved by the Ethics Committee of Anhui Medical University. Written informed consent was obtained from all participants.

### Covariates

Covariates were age, body mass index (BMI), sex (male, female), residential area (rural, urban), perceived family income (low, medium, high), cigarette use (yes, no), alcohol use (yes, no). During the past month, students who had smoked on at least 1 day were defined as cigarette users, and those who drank alcohol on at least 1 day were defined as alcohol users.

### Assessments instruments

#### PMPU

The Self-rating Questionnaire for Adolescent Problematic Mobile Phone Use developed by Tao et al. [17] was used to evaluate PMPU. This questionnaire comprises 13 items on three dimensions: withdrawal symptoms, craving, and physical and mental health status. Example items include: “I need to spend more time on my mobile phone to be satisfied” and “My leisure activities are reduced due to the time I spend on my mobile phone.” Responses to each item are on a five-point Likert scale from 1 to 5 (Not true at all, Slightly true, Moderately true, Strongly true, and Extremely true). Total scores range from 13 to 65, with higher scores reflecting more problematic use. The 75th percentile was used as the cut-off point; therefore, students with scores ≥28 were defined as PMPU. The Cronbach’s alpha coefficients for waves 1 and 2 were 0.91 and 0.92, respectively.

#### PA

PA was assessed the question “During the past 7 days, on how many days were you physically active for a total of at least 60 minutes per day? (Add up all the time you spend in any kind of PA that increased your heart rate and made you breathe hard some of the time).” This question was selected from the Youth Risk Behavior Survey [20]. Exercising for at least 60 min was chosen as the cut-off point because the American Physical Activity Guidelines recommend at least 60 min of moderate to vigorous PA on most days of the week for children and adolescents to attain health benefits [21]. This study used the 75th percentile (3 days) as the cut-off point; PA no more than 2 days per week was defined as IPA, and at least 3 days was defined as sufficient PA (SPA).

#### Depression symptoms

Depression symptoms were assessed with the Center for Epidemiologic Studies Depression Scale (CES-D) [22], which contains 20 items that assess subjective feelings during the past week. Response options are on a four-point rating scale: 0 = rarely or never / < 1 day, 1 = some or a little of the time / 1–2 days, 2 = occasionally or a moderate amount of the time / 3–4 days, 3 = most or all of the time / 5–7 days. A total score greater than 19 indicates depression. This measure was treated as a binary outcome using cut-off points based on symptom severity. Cronbach’s alpha coefficients for this scale have been previously reported [3].

#### Joint effects of PMPU and PA

To evaluate the joint effects of PMPU and PA, joint scores were calculated. Students with no PMPU and SPA were scored as 0, those with PMPU and SPA were scored as 1, those with no PMPU and IPA were scored as 1, and those with PMPU and IPA were scored as 2.

#### Increased depressive symptoms

Increased depressive symptoms were characterized as: participants who already had depression symptoms at wave 1, but had a higher CES-D score (even if only one point higher) in wave 2 than wave 1; and participants who don’t have depression symptoms (CES-D scores were below the CES-D cutoff) in wave 1 but have depression symptoms (CES-D scores were above the cutoff) in wave 2.

### Statistical analysis

Chi-square analyses were performed to assess sample characteristics by depression symptoms in wave 1 and increased depressive symptoms in wave 2. Totally 6 binary logistic regression models were run in this study. Four binary logistic regression models were used to calculate the crude OR and adjusted OR to examine the associations between PMPU (wave 1), PA (wave 1) and depression symptoms in waves 1 and 2 respectively. The dependent variable was depression symptoms and independent variables were PMPU, PA and Interaction effects of PMPU and IPA. Covariates were age, BMI, sex, residential area, perceived family income, cigarette use, and alcohol use for the model which calculate the adjusted OR. Another 2 binary logistic regression models were used to examine associations between PMPU (wave 1), PA (wave 1), and increased depressive symptoms. One was used to calculate the crude OR and another one was used to calculate the adjusted OR. For these two models, the dependent variable was increased depressive symptoms and independent variables were PMPU, PA and joint effects of PMPU and IPA. Covariates were age, BMI, sex, residential area, perceived family income, cigarette use, and alcohol use.

*P* < 0.05 was considered statistically significant. Statistical analyses were performed with SPSS V.23.0 (SPSS, version 23.0; SPSS, Inc., Chicago, IL, USA).

## Results

### Sample characteristics

The characteristics of the 2134 participating students (43% males, 57% females) are presented in Table 1. Participants’ mean age was 19.25 years (standard deviation 1.42 years). In total, 609 (28.5%) students reported PMPU, 1380 (64.7%) reported IPA, and 422 (19.8%) reported PMPU and IPA. Both depression symptoms (wave 1, 19.0% females, 15.3% males, *P* < 0.05) and increased depressive symptoms (11.1% females, 8.2% males, *P* < 0.05) were more common in females than males. Depression symptoms and increased depressive symptoms were also more common in students with PMPU and high scores for PMPU/PA joint effects. Students that reported IPA were more likely to be depressed in wave 1, but did not show increased depressive symptoms in wave 2. There were no significant differences in depression symptoms or increased depressive symptoms by residential area, perceived family income, and cigarette use. However, students who were recent alcohol drinkers showed lower depression symptoms (wave 1) and increased depressive symptoms (wave 2) than those who were not recent alcohol drinkers.

**Table 1:** Characteristics of the sample among Chinese college students [n (%)/M ± SD]

Variables	Overall	Depression symptoms (wave 1)			Increased depressive symptoms		
		Yes	No	*t/χ*^2^	Yes	No	*t/χ*^2^
Age	19.25 ± 1.42	19.41 ± 1.56	19.22 ± 1.39	2.37*	19.28 ± 1.55	19.25 ± 1.41	0.29
BMI	20.24 ± 2.40	20.11 ± 2.31	20.27 ± 2.42	1.16	20.09 ± 2.45	20.25 ± 2.39	0.96
Sex				5.02*			5.01*
Male	917 (43%)	140 (15.3%)	777 (84.7)		75 (8.2%)	842 (91.8)	
Female	1217 (57%)	231 (19.0%)	986 (81.0)		135 (11.1%)	1082 (88.9)	
Residential area				2.05			3.23
Rural	1217 (57%)	224 (18.4%)	993 (81.6)		132 (10.8%)	1085 (89.2)	
Urban	917 (43%)	147 (16.0%)	770 (84.0)		78 (8.5%)	839 (91.5)	
Perceived family income				2.11			0.49
Low	680 (31.9%)	130 (19.1%)	550 (80.9)		71 (10.4%)	609 (89.6)	
Medium	1348 (63.2%)	224 (16.6%)	1124 (83.4)		128 (9.5%)	1220 (90.5)	
High	106 (5.0%)	17 (16.0%)	89 (84.0)		11 (10.4%)	95 (89.6)	
Cigarette use				0.88			1.58
NO	1639 (76.8%)	278 (17.0%)	1361 (83.0)		154 (9.4%)	1485 (90.6)	
Yes	495 (23.2%)	93 (18.8%)	402 (81.2)		56 (11.3%)	439 (88.7)	
Alcohol use				4.74*			6.71*
No	938 (44%)	182 (19.4%)	756 (80.6)		110 (11.7%)	828 (88.3)	
Yes	1196 (56%)	189 (15.8%)	1007 (84.2)		100 (8.4%)	1096 (91.6)	
PMPU							
No	1525 (71.5%)	144 (9.4%)	1381 (90.6)	234.71***	117 (7.7%)	1408 (92.3)	28.32***
Yes	609 (28.5%)	227 (37.3%)	382 (62.7)		93 (15.3%)	516 (84.7)	
PA							
Sufficient	754 (35.3%)	109 (14.5%)	645 (85.5)	6.96**	66 (8.8%)	688 (91.2)	1.55
Insufficient	1380 (64.7%)	262 (19.0)	1118 (81.0)		144 (10.4%)	1236 (89.6)	
Joint effects of PMPU and IPA							
0	567 (26.6%)	43 (7.6%)	524 (92.4)	170.88***	39 (6.9%)	528 (93.1)	22.18***
1	1145 (53.7%)	167 (14.6%)	978 (85.4)		105 (9.2%)	1040 (90.8)	
2	422 (19.8%)	161 (38.2%)	261 (61.8)		66 (15.6%)	356 (84.4)	

*P < 0.05, **P < 0.01, ***P < 0.001

### Effect of PMPU, IPA, and PMPU/PA joint effects on depression symptoms

In wave 1, 609 (28.5%) students reported PMPU, 1380 (64.7%) reported IPA, and 422 (19.8%) reported PMPU and IPA. The binary logistic regression models showed associations of PMPU, IPA, and PMPU/IPA joint effects with depression symptoms (Table 2). Students who reported PMPU showed a positive correlation with depression symptoms in both wave 1 (odds ratio [*OR*] 5.76, 95% confidence interval [*CI*]: 4.52, 7. 36) and wave 2 (*OR* 3.20, 95% *CI*: 2.47, 4.15). IPA was positively associated with depression symptoms in wave 1 (*OR* 1.40, 95% *CI*: 1.09, 1.79) but not wave 2 (*OR* 1.24, 95% *CI*: 0.95, 1.62). Additionally, participants who reported PMPU and IPA were more likely to have depression symptoms than those who only reported PMPU or IPA in waves 1 (*OR* 7.36, 95% *CI*: 5.09, 10.66) and 2 (*OR* 3.74, 95% *CI*: 2.56, 5.48).

**Table 2:** The joint effects of problematic mobile phone use, physical activity (wave 1) and depression symptoms (wave 1 and 2)

	Depression symptoms					
	Wave 1			Wave 2		
PMPU	*N* (%)	Crude *OR* (*CI* 95%)	Adjusted *OR* (*CI* 95%) ^a^	*N* (%)	Crude *OR* (*CI*95%)	Adjusted *OR* (*CI*95%) ^a^
No	144 (9.4%)	1.00	1.00	139 (9.1%)	1.00	1.00
Yes	227 (37.3%)	5.70 (4.49,7.23)***	5.76 (4.52,7. 36)***	153 (25.1%)	3.35 (2.60,4.31)***	3.20 (2.47,4.15)***
PA	*N* (%)	Crude *OR* (*CI* 95%)	Adjusted *OR* (*CI* 95%)	*N* (%)	Crud*e OR* (*CI*95%)	Adjusted *OR* (*CI*95%)
Sufficient	109 (14.5%)	1.00	1.00	92 (12.2%)	1.00	1.00
Insufficient	262 (19%)	1.39 (1.09,1.77)**	1.40 (1.09,1.79)**	200 (14.5%)	1.22 (0.94,1.59)	1.24 (0.95,1.62)
Joint effects of PMPU and IPA						
0	43 (7.6%)	1.00	1.00	44 (7.8%)	1.00	1.00
1	167 (14.6%)	2.08 (1.46,2.96)***	1.99 (1.40,2.84)***	143 (12.5%)	1.70 (1.19,2.42)**	1.70 (1.19,2.44)**
2	161 (38.2%)	7.52 (5.20,10.86)***	7.36 (5. 09,10.66)***	105 (24.9%)	3.94 (2.70,5.75)***	3.74 (2.56,5.48)***

^a^Adjusted for age, BMI, sex, residential area, perceived family income, cigarette use, alcohol use

*P < 0.05, **P < 0.01, ***P < 0.001

### Interactions of PMPU, IPA, and increased depressive symptoms

Table 3 shows the interactions of PMPU (wave 1), IPA (wave 1), and increased depressive symptoms. The adjusted regression models showed no differences between PA and increased depressive symptoms. However, compared to those without PMPU, students with PMPU were positively associated with increased depressive symptoms (*OR* 2.05, 05% *CI*: 1.52, 2.75). Students with PMPU and IPA showed a higher *OR* (*OR* 2.36, 95% *CI*: 1.55, 3.60) for increased depressive symptoms than those who only reported PMPU or IPA.

**Table 3:** Interactions of problematic mobile phone use, physical activity and increased depressive symptoms

	Increased depressive symptoms		
	*N* (%)	Crude *OR* (*CI* 95%)	Adjusted *OR* (*CI* 95%) ^a^
PMPU			
No	117 (7.7%)	1.00	1.00
Yes	93 (15.3%)	2.17 (1.62,2.90)***	2.05 (1.52,2.75)***
PA			
Sufficient	66 (8.8%)	1.00	1.00
Insufficient	144 (10.4%)	1.21 (0.89,1.65)	1.23 (0.90,1.67)
Joint effects of PMPU and IPA			
0	39 (6.9%)	1.00	1.00
1	105 (9.2%)	1.37 (0.93,2.00)	1.37 (0.93,2.02)
2	66 (15.6%)	2.51 (1.65,3.81)***	2.36 (1.55,3.60)***

^a^Adjusted for age, BMI, sex, residential area, perceived family income, cigarette use, alcohol use

*P < 0.05, **P < 0.01, ***P < 0.001

## Discussion

Based on the 2134 college students involved in this follow-up study, we examined the simultaneous association between PMPU and IPA and depression symptoms. The results showed there was a positive joint effect of PMPU and IPA on depression symptoms and increased depressive symptoms at follow-up.

In this study, the prevalence of PMPU was 28.5%, which was higher than that reported in previous studies involving Chinese college students (8.99–28.2%) [19, 23]. The prevalence of IPA in this study was 64.7%, which was markedly higher than rates reported in the United States (35.1%) [24], Finland (17.0%) [25], and Singapore (7.2%) [26]. Additionally, we found that 17.4% of participating students had depression symptoms, which was higher than the 3.59% [27] reported for the general population in China but lower than the 30.6% reported for college students worldwide [28]. The depression rate in this sample of college students was 3.85 times higher than the rate in the general population, which suggests that this issue needs more attention in China.

Many studies focused on children and adolescents have confirmed that PA has beneficial effects on depression symptoms [29–33]. High-level exercise is also more efficacious in reducing depression symptoms. However, the association in our study was not obvious. Babyak et al. noted that PA can be used as a therapeutic method for acute and chronic depression, and was comparable with antidepressant treatments [34]. The “endorphin hypothesis” concerning PA as a treatment for depression symptoms is widely accepted. This hypothesis suggests that mood states are dependent on endorphin secretion and exercise augments endorphin secretion in the brain, which can reduce pain and cause general euphoria, thereby reducing anxiety and depression levels [35, 36]. Swathi et al. reviewed relevant studies and stated that neural mechanisms may also mediate the positive effects of exercise on depressive symptoms [37].

PMPU has also been shown to be associated with sleep quality [19] and mental health, including psychopathological symptoms, anxiety, depression, and chronic stress [19, 23, 38, 39]. However, the nervous system has been found to be most sensitive to the effects of mobile phones [40, 41]. For example, harmful effects from mobile phone signals have been observed during electroencephalogram testing [42, 43]. Our findings from the follow-up study also supported the conclusion that PMPU was positively associated with increased depressive symptoms.

IPA and PMPU are considered universally coexisting behaviors among college students, although research on the combined effects of IPA and PMPU on depression is lacking. Mobile phones may positively influence our behavior through providing activity reminders, aiding in tracking and monitoring PA, and providing entertainment during PA, but also negatively affect behaviors such as promoting sedentary screen time. Addictive mobile phone users tend to get less PA, and IPA is often associated with mobile phone use [44]. However, Matthew et al. found that mobile phone users tended to have more sitting time but were more likely to meet PA recommendations than nonusers in a Mexican American cohort [45]. In the present study, participants with PMPU were more common in the IPA group than the high PA group (*n* = 422, 69.3% vs. *n* = 187, 30.7%; *P* < 0.01). PA maybe affected by PMPU, and PMPU and IPA are negatively associated with depression symptoms. In view of these relationships, we introduced a new variable “PMPU/PA joint effects.” The group with high PMPU/PA joint effect scores had a greater *OR* for depression symptoms and increased depressive symptoms compared with those with low scores and PMPU or IPA alone, which indicated a positive joint effect of PMPU and IPA on depression symptoms. This suggested that the joint effect of PMPU and IPA may promote depression symptoms among college students.

Our study revealed that active students without PMPU had fewer symptoms of depression than their sedentary peers with PMPU. Students with PMPU and IPA were more likely to have severe depressive symptoms. PA is closely related with mobile phone use. While emphasizing the increase of students’ physical activity, they should also emphasize the control of students’ mobile phone use. In other words, inhibiting PMPU behavior and increasing PA may more conducive to prevent depression symptoms. Therefore, it is important to encourage increased PA and less mobile phone use among students to promote their physical and mental health.

There were several limitations in the present study. First, there was only one follow-up assessment, and the total study period was only 6 months. Second, the self-reported data might have introduced recall and reporting biases. Finally, only one university was involved in this study, which could affect the generalizability of the findings. However, the large sample in our study may mitigate these limitations. Furthermore, this was the first time the association between joint of PMPU and PA with depression symptoms was explored among college students.

## Conclusions

In this study, PMPU was significantly associated with depression symptoms and increased depressive symptoms at follow-up. IPA showed slight effects on depression symptoms in wave 1. This study indicates there may be a joint effect of PMPU and IPA on depression symptoms and increased depressive symptoms. Depression symptoms could be prevented by controlling the use of mobile phones and increasing PA. These results may suggest that PMPU is an important contributing factor for depression in college students, and IPA could be a synergistic factor.

## Data Availability

The datasets used and/or analysed during the current study are available from the corresponding author on reasonable request.

## Abbreviations

BMI: Body mass index
CES-D: Center for Epidemiologic Studies Depression Scale
CI: Confidence intervals
IPA: Insufficient physical activity
OR: Odds ratios
PA: Physical activity
PMPU: Problematic mobile phone use
SPA: Sufficient PA
